# Cross-domain interactions confer stability to benthic biofilms in proglacial streams

**DOI:** 10.3389/frmbi.2023.1280809

**Published:** 2024-01-11

**Authors:** Susheel Bhanu Busi, Hannes Peter, Jade Brandani, Tyler J. Kohler, Stilianos Fodelianakis, Paraskevi Pramateftaki, Massimo Bourquin, Grégoire Michoud, Leïla Ezzat, Stuart Lane, Paul Wilmes, Tom J. Battin

**Affiliations:** ^1^ UK Centre for Ecology & Hydrology (UKCEH), Wallingford, Oxfordshire, United Kingdom; ^2^ Systems Ecology Group, Luxembourg Centre for Systems Biomedicine, University of Luxembourg, Esch-sur-Alzette, Luxembourg; ^3^ River Ecosystems Laboratory, Alpine and Polar Environmental Research Center, Ecole Polytechnique Fédérale de Lausanne, Lausanne, Switzerland; ^4^ Department of Ecology, Faculty of Science, Charles University, Prague, Czechia; ^5^ MARBEC, Université de Montpellier, CNRS, Ifremer, IRD, Montpellier, France; ^6^ Institute of Earth Surface Dynamics (IDYST), University of Lausanne, Lausanne, Switzerland; ^7^ Department of Life Sciences and Medicine, Faculty of Science, Technology and Medicine, University of Luxembourg, Esch-sur-Alzette, Luxembourg

**Keywords:** glacier-fed streams, cross-domain interactions, networks, community fragmentation, microbiome

## Abstract

Cross-domain interactions are an integral part of the success of biofilms in natural environments but remain poorly understood. Here, we describe cross-domain interactions in stream biofilms draining proglacial floodplains in the Swiss Alps. These streams, as a consequence of the retreat of glaciers, are characterised by multiple environmental gradients and perturbations (e.g., changes in channel geomorphology, discharge) that depend on the time since deglaciation. We evaluate co-occurrence of bacteria and eukaryotic communities along streams and show that key community members have disproportionate effects on the stability of community networks. The topology of the networks, here quantified as the arrangement of the constituent nodes formed by specific taxa, was independent of stream type and their apparent environmental stability. However, network stability against fragmentation was higher in the streams draining proglacial terrain that was more recently deglaciated. We find that bacteria, eukaryotic photoautotrophs, and fungi are central to the stability of these networks, which fragment upon the removal of both pro- and eukaryotic taxa. Key taxa are not always abundant, suggesting an underlying functional component to their contributions. Thus, we show that there is a key role played by individual taxa in determining microbial community stability of glacier-fed streams.

## Introduction

Biofilms represent the dominant microbial lifestyle in streams and rivers (Battin et al., 2016). These matrix-enclosed microbial communities colonise sediment surfaces, alongside the surface of benthic rocks and plants, and regulate critical ecosystem processes (Battin et al., 2016). Stream biofilm communities are highly diverse, harbouring members from all domains of life, including prokaryotes and various microeukaryotes. This biodiversity enclosed within streams fosters interactions, such as those between bacterial heterotrophs, algae, and fungi. The bacterial and eukaryotic photoautotrophic interactions, the latter of which includes algae, are likely due to metabolic exchanges, whereby algal exudates serve as a source of organic matter for bacterial heterotrophs (Kaplan and Bott, 1989; Wagner et al., 2017). The fungi, meanwhile, may play important roles where fungal metabolites are utilised by distinct bacterial taxa such as Burkholderia (Stopnisek et al., 2016). Furthermore, they are also capable of parasitizing algae, in the case of Chytridiomycetes, to release carbon for bacterial utilisation (Klawonn et al., 2021).

In plants and animals, elevated biodiversity and related biotic interactions can contribute to community stability (McCann, 2000). Yet, studying biotic interactions within complex microbial communities is not a trivial task. For instance, signalling molecules have been suggested as a proxy for identifying interactions among microorganisms (Braga et al., 2016). However, such approaches have remained largely limited to model systems (Vetsigian et al., 2011; Lee and Zhang, 2015), e.g., communities of *Streptomyces, Pseudomonas* and *Vibrio* spp. Given the multitude of interacting taxa and the small spatial scales at which interactions occur, the direct observation and quantification of individual microorganisms in stream biofilms is not possible to this date. Instead, patterns of taxa co-occurrence across samples can be used to infer microbial interactions, niches, and key taxa (Faust and Raes, 2012; Berry and Widder, 2014). Despite limitations stemming from the notion that co-occurrence networks are non-empirical and derived from correlations, co-occurrence patterns are often useful for assessing ecological networks and obtaining insights into the organisation of microbial communities (Faust and Raes, 2012; Layeghifard et al., 2017). Co-occurrence networks further allow the exploration of emergent properties, such as the density of interactions, clusters of interacting taxa or the stability of networks against fragmentation. For example, studying bacterial co-occurrences across a dendritic stream network, Widder et al. found evidence for the role of spatial and hydrological processes in shaping co-occurrence network structure and stability (Widder et al., 2014). More recently, Ma et al. highlighted the interconnected patterns across microbiomes in various environments, emphasising the critical impact of microbial interactions on community assembly processes (Ma et al., 2020).

The environment of glacier-fed streams (GFS) is harsh. Low water temperature, coupled with high turbidity, oligotrophy, and snow- and ice-cover over extended periods of the year collectively contribute to this harshness. Unstable stream channels resulting from high turbulence and sediment loads further contribute to challenging conditions, making it difficult for benthic biofilms to establish (Roncoroni et al., 2023). Depending on local topography and geomorphology, GFS may develop into extensive floodplains, with dynamic braided channels exhibiting channel migration on a diel basis (Roncoroni et al., 2023). As distance increases downstream, harshness tends to decrease as channels consolidate and pioneering vegetation stabilises channels (Miller and Lane, 2019). Channel stabilisation increases the habitability of GFS, making these ecosystems more hospitable for benthic biofilms (Roncoroni et al., 2023). Furthermore, towards the edges of the proglacial floodplain, non-glacial tributary streams (TRIB), predominantly fed by groundwater and snowmelt, drain adjacent elevated terrasses that ultimately discharge into the glacier-fed mainstem (Brown et al., 2007). Given the lack of connectivity with the glacier, the TRIB environment is therefore more stable compared to GFS (Roncoroni et al., 2023), with potential consequences for biofilm structure and function (Freimann et al., 2013; Michoud et al., 2023). In fact, despite their close spatial proximity, GFS and TRIB streams host biofilms that differ in their biomass, composition, and diversity, as well as functional potential (Freimann et al., 2013; Brandani and Busi, 2022). It is predicted that as glaciers shrink, GFS will gradually transition into streams fed by groundwater and snowmelt, hence becoming more alike TRIB (Milner et al., 2017; Kohler et al., 2022). However, the consequences of this environmental change for microbial life remain poorly understood.

Brandani et al. recently reported on the complexity of GFS benthic biofilms in the proglacial floodplains of the Swiss Alps (Brandani and Busi, 2022). For instance, alongside bacteria, eukaryotic photoautotrophs such as Ochrophytes (algae) are a major component of these streams. Additionally, fungi such as Chytridiomycetes play key roles in microbial interactions, being involved in 12% of the overall interactions within these streams (Brandani and Busi, 2022). However, the role of taxa within these complex networks in influencing and maintaining community composition have not been resolved. Here, we investigated the properties of cross-domain co-occurrence networks of benthic biofilms in GFS and TRIB with different deglaciation histories in two proglacial floodplains in the Swiss Alps. We focused on putative interactions between bacteria, eukaryotic photoautotrophs, and parasitic fungi. Eukaryotic photoautotrophs are indeed important to stream biofilms as their exudates constitute an energy source for heterotrophic bacteria (e.g. during algal blooms (Kaplan and Bott, 1989), in the presence of light (Battin et al., 2003; Wagner et al., 2017)), while fungi can parasitize algae, thereby affecting microbial carbon flow (Klawonn et al., 2021). Therefore, we posit eukaryotic photoautotrophs and fungi as keystone taxa that take a central role, defined here based on network centrality measures, in maintaining network structure, and further hypothesise that the apparent stability of co-occurrence networks in GFS and TRIB changes along downstream and lateral gradients of deglaciation histories and environmental stability. To evaluate these roles, we assessed the stability of cross-domain co-occurrence networks upon removal of keystone taxa. We also investigated the variance in bacterial community composition that can be explained by specific bacterial and eukaryotic keystone taxa, subsequently contrasting this to the variance that can be explained by environmental differences among sites.

## Materials and methods

### Sample collection

Benthic biofilms were collected from stream sediments (0 - 5 cm depth) near to the glacier snout and extending to the floodplain’s outlet in various TRIB and GFS stream reaches within the Otemma Glacier (Otemma; 45° 56’ 08.4” N, 7° 24’ 55.1” E) and Val Roseg Glacier (Val Roseg; 46° 24’ 21.1” N, 9° 51’ 55.1” E) floodplains in Switzerland. In each reach, we collected sandy sediments (0.25 - 3.15 mm) with flame-sterilised sieves and spatulas. Sediments samples were transferred to sterile vials, immediately frozen in the dark on dry ice in the field, and stored at -80°C in the laboratory until analysis (all of which were completed within a few months). Samples were collected during early (June/July) and late (August/September) summer (Brandani and Busi, 2022) in 2019. As shown previously, the two sample periods did not show differences in terms of community composition and structure (Brandani and Busi, 2022). For water, i.e. DOC and major ion measurements, samples were refrigerated, while nutrient samples were frozen at -20 C. Samples for the analysis of ions were sterile-filtered and kept at 4C until processing within a few weeks. Samples for the analysis of DOC were GF/F filtered, kept at 4C in the dark and analysed immediately upon return to the laboratory (i.e. within 48h after sampling). Study reaches were categorised into GFS or TRIB depending on their connectivity to glacier runoff based on visual field observations, drone-based imagery, and physicochemical characteristics (Brandani and Busi, 2022). The physicochemical parameters were established previously as part of the study characterising the diversity of the pro- and eukaryotic communities (Brandani and Busi, 2022). Overall, a total of 136 samples (GFS: 50; TRIB: 86) were collected across both floodplains. The sampling sites, indicating pre-2000 (UP) versus post-2000 (DOWN), from GFS and TRIB across Otemma and Val Roseg are depicted in **Supplementary Figure 1**. These included 68 samples each for the Otemma Glacier and Val Roseg Glacier floodplains, with the exact breakdown of these samples into GFS (Otemma:20; Val Roseg: 30) and TRIB (Otemma:48; Val Roseg: 38) including metadata listed in **Supplementary Table 1**.

### Deglaciation histories

We identified past glacier extents from historic orthophotos and maps using SWISSIMAGE journey through time (Rickenbacher, 2020), and the GLIMS glacier inventory (Raup et al., 2007). These extents were compared with GLAMOS (Linsbauer et al., 2021) frontal variation measurements to verify glacial re-advances. The year of latest glaciation was thus interpolated for each sample site, which provided the longitudinal deglaciation history. We further split the reaches of the floodplain into those which were already deglaciated in the year 2000 (post-2000 or DOWN) and those still glaciated in 2000 (pre-2000 or UP; **Supplementary Figure 1A**). A lateral gradient (relative to the GFS mainstem) is also given by the TRIB streams that drain the adjacent terraces on the margins of the proglacial floodplains.

### Benthic algal biomass

Benthic algal biomass was estimated as chlorophyll *α* (**Supplementary Table 2**) using a modified ethanol extraction protocol (Kohler et al., 2020), processed within a few months after collection. For this, sediment (ca. 2 g) samples were treated with 5 ml of 90% EtOH and then placed in a hot water bath (78°C, 10 min), followed by an incubation in the dark (4°C, 24 h). They were thereafter vortexed, centrifuged, and the supernatant read on a plate reader at 436/680 nm (excitation/emission). Chlorophyll *α* concentrations were inferred from a spinach standard and normalised by the sediment dry mass (DM).

### Metabarcoding library preparation and sequencing

A previously established protocol (Busi et al., 2020) utilising phenol-chloroform was used for DNA extraction from benthic sediments (ca. 0.5 g). After initial processing, the DNA samples were diluted to a final concentration of ≤ 2-3 ng/µl. For the 16S rRNA gene metabarcoding analyses, we used the methodology previously described in Fodelianakis et al. (Fodelianakis et al., 2022), where the V3-V4 hypervariable region of the 16S rRNA gene were targeted with the 341F/785R primers. This was done in line with the 16S library preparation Illumina guidelines for the MiSeq system. The eukaryotic 18S rRNA gene metabarcoding library preparation was performed similarly but using the TAReuk454F-TAReukREV3 primers (Stoeck et al., 2010). Based on the MiSeq manufacturer’s protocol, amplicon libraries were prepared where a second PCR was used to add dual indices to the purified amplicon PCR products. This allowed for extensive multiplexing of samples on a single sequencing lane of the MiSeq (Illumina) platform after quantification and normalisation. Samples were subsequently sequenced using a 300-base paired-end protocol in the Lausanne Genomic Technologies Facility (Switzerland).

### Metabarcoding analyses

For the 16S and 18S rRNA metabarcoding data analyses, a combination of Trimmomatic v0.36 (Bolger et al., 2014) and QIIME2 v.2020.8 (Bolyen et al., 2019) were used with the latest SILVA database (Quast et al., 2013) v138.1 for taxonomic classification of the gene amplicons, i.e. 16S rRNA and 18S rRNA. From the 16S rRNA amplicon dataset, non-bacterial amplicon sequence variants (ASVs), i.e., archaea, chloroplasts, and mitochondria, were removed from all downstream analyses. The dataset was not rarefied for the analyses owing to the saturation of sequencing curves (Brandani and Busi, 2022) and also based on our previous work where rare taxa play important roles in GFS community structure (Wilhelm et al., 2014). Additionally, the rationale behind discarding the archaeal reads was that the primers used were not designed, and are therefore not optimal, for detecting all lineages of archaea (Bahram et al., 2019). A total of 192 sample libraries were generated for the 16S rRNA sequencing and paired-end sequencing produced a total of 17,200,512 reads, with an average of 89,586 reads per sample. For the 18S rRNA amplicon dataset, a total of 157 amplicon sequence libraries from sediment samples were generated. Meanwhile, singletons and ASVs observed only once were discarded. The paired end 18S rRNA sequencing generated a total of 10,837,518 reads, with an average of 64,127 reads per sample. The 18S ASVs were further clustered into operational taxonomic units (OTUs) based on a 97% identity threshold using the *de novo* clustering method in *vsearch*, which has been implemented in QIIME2. This approach was used in order to avoid the overestimation of diversity driven by a high copy number of 18S in eukaryotic cells (Brandani and Busi, 2022). For the analyses, photoautotrophic eukaryotes along with fungi and protists were retained in the 18S rRNA amplicon dataset, given their contributions to community composition as highlighted in our previous work (Battin et al., 2016). Consequently, other non-phototrophic eukaryotes were discarded from the downstream analyses. The 18S rRNA dataset was also not rarefied, and any singletons/OTUs observed in only one sample were removed from downstream analyses, resulting in a dataset of 18S rRNA eukaryotic photoautotrophs and fungi with 429 OTUs. For our downstream analyses (i.e., co-occurrence), it was critical to include paired datasets. This was also a limitation of SpiecEasi (Kurtz et al., 2015), where only paired samples can be included to run pro- and eukaryotic network analyses. To address this, only 136 samples with paired 16S and 18S data were used for downstream analyses.

### Co-occurrence networks

To study potential interactions between bacteria, eukaryotic photoautotrophs, and fungi, co-occurrence network analyses were performed with samples meeting specific criteria. These included: 1) the presence of both 16S and 18S sequence data for each sample, and 2) samples had to be categorised the same way across both samplings to ensure replicability (i.e., either designated as GFS or TRIB for both samplings as described by Brandani et al. (Brandani and Busi, 2022). Due to the dynamic nature of proglacial streams, GFS channels tend to meander and migrate, leaving some sites dry, under the influence of TRIB, or infiltrating TRIB streams. Hence, this approach was adopted to avoid potential confounders.

Subsequently, to reduce the noise and overall computational effort, any ASVs found in less than 5% of the samples were discarded from the 16S dataset for the co-occurrence networks. Prior to setting a threshold of 5%, the total number of ASVs retained in the datasets at 1, 5 and 10% thresholds was assessed to reduce noise and computational burden. From a total of 25,307 and 26,912 ASVs in Otemma and Val Roseg, respectively, only 5,268 and 5,216 ASVs were retained at 10%. To avoid exclusivity to only highly-prevalent taxa, we chose the 5% cutoff, where, 17,825 and 16,512 ASVs were respectively retained in Otemma and Val Roseg (**Supplementary Table 3**).

Co-occurrence networks between 16S and 18S (i.e., bacteria, eukaryotic photoautotrophs and fungi) were constructed using an ensemble of the distance matrices created from SparCC (Friedman and Alm, 2012), Spearman’s correlation (Hauke and Kossowski, 2011), and SpiecEasi (Kurtz et al., 2015) where the networks were constructed using the Meinshausen and Bühlmann (mb) method (Meinshausen and Bühlmann, 2006). Networks were constructed across reaches, for pre-2000 and post-2000segments separately, and across GFS and TRIB for both Otemma and Val Roseg floodplains. Since our analyses are based on amplicon sequence data alone, we focused on the positive interactions across domains to assess potential mutualism within the microbiome. While reports suggest that negative interactions are indicative of co-exclusion mechanisms, especially in human microbiomes (Faust et al., 2012), the paucity of information available, especially in poorly characterised ecosystems may be insufficient to establish via amplicon sequencing data.

To detect communities in the network analyses, we used the Louvain clustering algorithm (Ghosh et al., 2018), removing clusters with less than five nodes. Herein, each community is defined as nodes within the graph with a higher probability of being connected to each other than to the rest of the network. Following this, we calculated network topology measures, including number of nodes and edges, number of clusters, diameter, edge-density, and modularity. These refer to the overall structure of the network such as the number of constituent entities (nodes), their connections (edges), overall length of connections (diameter), and independent cliques (clusters). The adjacency matrix was visualised using the *igraph* package (Csardi and Nepusz, 2006) in R v4.0.3 (R Core Team, 2021). Centrality measures, degree and betweenness, were also estimated per node, using the *igraph* v1.3.4 package. The fragmentation (*f*) of the network was determined as the percentage of the number of disconnected subgraphs over the overall nodes in each network (Widder et al., 2014). Fragmentation was estimated iteratively by the removal of each keystone taxa, i.e., top 10 nodes with both a high degree and a high betweenness in each graph. This information was further used for the subsequent generation of network topologies such as the number of clusters following the initial Louvain clustering of the network.

### Community analyses

To explore the role of keystone taxa in structuring biofilm communities, we used constrained ordinations (db-RDA, R function vegan:capscale) using Bray-Curtis distances. In contrast to unconstrained ordination, constrained, or canonical ordination, resolves only the variation that can be explained by the constraints. We first employed a forward selection strategy (vegan:ordistep) to identify sets of non-redundant and significant (*p* < 0.01) bacterial and eukaryotic keystone taxa that we used as constraints for RDA. We subsequently used the explained variance in the constrained ordination as a measure of how important bacterial and eukaryotic keystone taxa are for explaining bacterial community composition in the two floodplains. Prior to db-RDA, Wisconsin-double standardisation was applied to the ASV counts. The relative abundances of keystone taxa were inspected for multicollinearity and were provided as constraints for stepwise model creation (using 999 permutations). Model significance was evaluated for each RDA axis and explained variance of the constraints was extracted. To contrast variance in bacterial community composition that could be explained by keystone taxa, we performed a similar analysis using environmental parameters. For this, important environmental parameters including pH, water temperature, specific conductivity, dissolved oxygen (DO), turbidity and major ions and nutrients (obtained from Brandani and Busi, 2022) were first standardised (log-transformed) and then supplied to forward selection in db-RDA as described above.

### Data analysis

All statistical analyses were performed in R v4.0.3. The *ggplot2* (Wickham, 2011) package was used for generating plots in R, while *patchwork* (https://github.com/thomasp85/patchwork) and Adobe Illustrator were used to arrange the figures as displayed.

## Results

### Cross-domain interactions underlie stream community structure in proglacial floodplains

**Figure 1:**
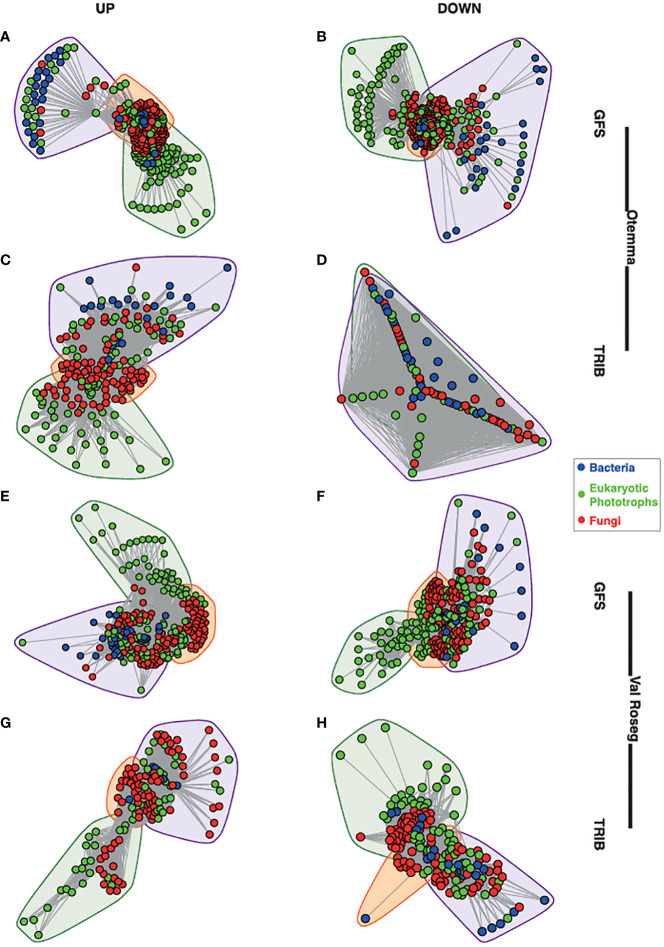
Network structure of glacier-fed streams and tributary streams. The overall structure of the cross-domain networks from the glacier-fed streams (GFS) and non-glacial tributaries (TRIB) are depicted. **(A)** GFS from the pre-2000reaches at Otemma, **(B)** GFS from the pre-2000reaches at Otemma, **(C)** TRIB from the post-2000 reaches at Otemma, **(D)** TRIB from the pre-2000reaches at Otemma. From the Val Roseg glacier, the network structures are depicted as follows: **(E)** GFS from the pre-2000reaches, **(F)** GFS from the post-2000 reaches, **(G)** TRIB from the pre-2000reaches, **(H)** TRIB from the post-2000 reaches. Each node represents a single amplicon sequence variant (ASV), and the lines represent the edges between them, while the colours indicate bacteria, eukaryotic photoautotrophs (indicated as phototrophs) and fungi. The convex hulls indicate clusters identified based on Louvain clustering of the overall network.

In both proglacial floodplains, GFS and TRIB harbour diverse microbial communities which include bacteria, fungi, and phototrophic eukaryotes (**Supplementary Figure 1**). Previously, we reported on the overall community structure, diversity and abundances of the various microbial components in these streams (Brandani and Busi, 2022), including the role of benthic primary producers in shaping the microbial communities (Michoud et al., 2023). Microalgae such as Chlorophyta, Charophyta, Cryptomonadales, and Ochrophyta form the majority of the phototrophic eukaryotes, which collectively contribute a higher diversity in TRIB compared to GFS (Brandani and Busi, 2022). We previously observed the richness and Pielou’s evenness indices were higher in TRIB compared to GFS. In light of this, we similarly found that bacterial communities were more diverse within the TRIB and dominated by Proteobacteria (Gamma- and Alphaproteobacteria), Bacteroidia, Cyanobacteria, and Verrucomicrobia. Based on covariation of taxa abundances across samples, we built co-occurrence networks. These networks were based on 1,090 nodes including bacteria, eukaryotic photoautotrophs, and fungi, with an average of 61,115 edges (interactions) connecting the nodes. The topological characteristics of the individual networks yielded similar metrics, reflected in density, modularity, assortativity and transitivity (**Supplementary Table 1**). In all networks, except Otemma post-2000, we identified three dense clusters of co-occurring taxa, one with a majority of eukaryotic photoautotrophs, another comprising mainly bacteria, and an intersecting third cluster composed of microbial eukaryotes including fungi and bacteria (**Figure 1**).

Next, we assessed the relative abundance of taxa present in the networks at the family level. Across both floodplains and stream types, we found that Acetobacteraceae were significantly overrepresented in networks constructed from pre-2000compared to post-2000reaches (two-way ANOVA, adj. *p* < 0.05, **Supplementary Figures 2A, B**, **3B**). On the other hand, Comamonadaceae were significantly overrepresented in post-2000networks (two-way ANOVA, adj. *p* < 0.05), especially in TRIB (**Supplementary Figures 2B** and **3B**). We also found that Chrysophyceae were overrepresented in pre-2000networks, while Diatomea decreased in pre-2000networks (two-way ANOVA, adj. *p* < 0.05) (**Supplementary Figures 2C, D** and **3C, D**). Chytridiomycota, parasitic fungi infecting algae (Klawonn et al., 2021), were prevalent in both GFS and TRIB networks, but their abundance did not significantly differ across pre-2000or post-2000sites. However, Zoopagomycota, also parasitic fungi (Spatafora et al., 2016), were considerably enriched in post-2000 reaches across stream types and floodplains (**Supplementary Figures 1E-F** and **3E, F**; adj. *p* < 0.05, Two-way ANOVA).

### Apparent stability of co-occurrence networks

Based on our observations of differential abundance patterns across stream types and deglaciation gradients, we further assessed the contributions of the individual taxa to the overall network. For this, we first identified potential keystone taxa within each network by identifying the top 10 nodes with both a high degree and a high betweenness in each network (**Supplementary Figures 4** and **5**). For example, taxa classified as Dikarya, Phragmoplastophyta, Chlorophyceae, Cryptomycota, and Diatomea, along with an ASV classified as Burkholderiales, were determined to be keystone taxa in the GFS network at the pre-2000segment of the Otemma Glacier floodplain (**Supplementary Figure 4A**). Conversely, at the post-2000 segment of the same floodplain, Burkholderiales, Phragmoplastophyta, Xanthophyceae, Chrysophceae, and Dikarya, for instance, were identified as keystone taxa. Similarly, in the pre-2000segment of the Val Roseg Glacier floodplain, Dikarya, Phragmoplastophyta, Gemmatales, Burkholderiales, Cryptomycota, and Diatomea, for instance, were identified as keystone taxa contributing to the network topology (**Supplementary Figures 5A, C**). Finally, we found various bacteria (e.g., Rhodobacterales, Sphingomonadales) and fungi (e.g., Chytridiomycota) to be keystone taxa in the post-2000 reaches within the Val Roseg floodplain (**Supplementary Figures 5B, 5D**).

**Figure 2:**
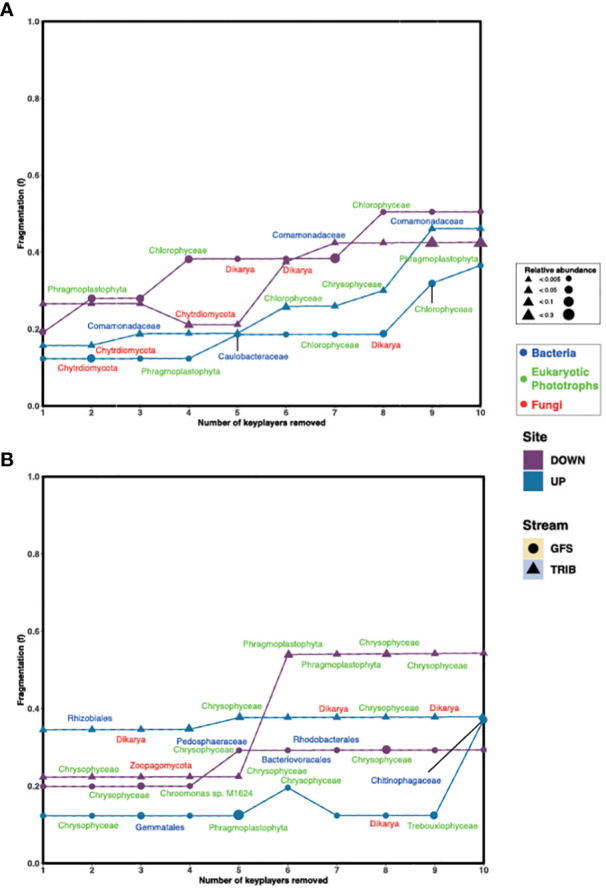
Keystone removal leads to fragmentation of the network. The change in fragmentation (*f*) for **(A)** Otemma and **(B)** Val Roseg are indicated in the line plots, where *f* was recalculated after each keystone (i.e., bacteria, eukaryotic photoautotroph or phototroph and fungi) was removed from the network. The size of the symbols indicates the relative abundance of the individual keystone taxa within the 16S or 18S data, respectively.

To further understand the role of the keystone taxa in community structure and their effect on apparent network stability, we first assessed network fragmentation upon their removal. For this, the numbers of clusters based on Louvain clustering were determined for each network, following which, a keystone was removed. The fragmentation (*f*) of the network was assessed before and after iterative removal of the top 10 keystone taxa. Interestingly, we found that in the Otemma Glacier floodplain (**Figure 2A**), the fragmentation of the networks constructed from the GFS in the post-2000 reaches, increased upon removal of two to three keystone taxa, while the TRIB fragmentation increased upon removal of five keystone taxa. The pre-2000networks, however, appeared more stable, where fragmentation occurred only upon removal of five or eight keystone taxa. In Val Roseg, especially in TRIB (**Figure 2B**), the overall fragmentation of the microbial network was higher (*f*_mean_=0.48) compared to GFS (*f*_mean_=0.18) upon removal of four or five keystone taxa.

**Figure 3:**
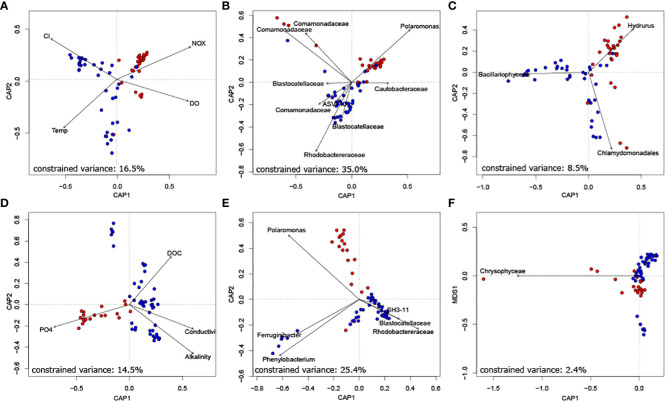
Bacterial keystone taxa well explain bacterial community composition. Constrained ordination of Val Roseg **(A–C)** and Otemma **(D–F)** floodplain samples revealed that bacterial keystone taxa **(B, E)**, as identified by their position in co-occurrence networks explained most of the variance in Bray-Curtis distance-based bacterial community composition. This outweighed the role of key environmental parameters **(A, D)** and of eukaryotic keystone taxa **(C, F)**.

Finally, we unravelled the role of keystone taxa for biofilm community composition. Constrained ordinations revealed that both bacterial as well as eukaryotic phototrophic keystone taxa can explain a substantial fraction of bacterial community dissimilarity at the floodplain scale (**Figure 3**). Specifically, the relative abundance of bacterial keystone taxa explained 35.0% and 25.4% of variance in bacterial community similarity in Val Roseg and Otemma, respectively. While eukaryotic keystone taxa appeared particularly important for explaining network stability, they played a minor role in explaining bacterial community composition (i.e., 8.5% and 2.4% of explained variance in Val Roseg and Otemma, respectively). This is surprising, particularly in relation to the variance in bacterial community composition that could be explained by environmental conditions, which accounted for a mere 16.5% and 14.5%, respectively. The retained environmental parameters, including streamwater temperature, nutrients and DOC concentration explain differences among TRIB and GFS bacterial communities.

## Discussion

Biotic interactions are a salient property of microbial communities, with evidence of cross-domain interactions reported from various ecosystems, including freshwaters (Sun et al., 2022; Wijewardene et al., 2022), oceans (Rowan-Nash et al., 2019), glaciers (Brown and Jumpponen, 2015), and icesheets (Perini et al., 2023). To date, such interactions have not been studied in proglacial stream biofilms. Our findings suggest that biotic interactions, as inferred from co-occurrence patterns, play a pivotal role in influencing the apparent stability of stream biofilm communities along deglaciation and environmental gradients. Although previous reports showed structural and functional differences of the biofilm communities dwelling in different stream types within proglacial floodplains (Freimann et al., 2013; Brandani and Busi, 2022), we found that the overall network topology was similar between both proglacial floodplains, stream types, and deglaciation gradients. This contrasts with our expectation of successional imprints owing to deglaciation on co-occurrence networks. On the one hand, biotic interactions may be established very early on during community succession in the streams that drain recently deglaciated terrain. Indeed, our sampling design covered the successional timescale of the past 20 (pre-2000sites) and 80 (post-2000) years. Furthermore, functional redundancies across clades may also contribute to the apparent similarity of cross-domain interaction networks. Functionally redundant taxa may transiently occupy the same position in interaction networks and therefore result in similar network topologies. However, additional work will be necessary to relate network topology, taxa position and stability with functional characteristics to substantiate this notion.

Cross-domain networks have the potential to reveal key associations between microbial taxa (Williams et al., 2014). We found that biofilms in GFS and TRIB draining recently deglaciated terrain (i.e., pre-2000sites) had relatively more stable networks compared to the post-2000 sites. This finding suggests that bacterial keystone taxa are important for the apparent stability of the cross-domain interaction networks of biofilms dwelling in these nascent stream ecosystems. Furthermore, our results reveal that not all keystone taxa are typically among the most abundant community members, suggesting that low abundance taxa may also play important roles in stabilising microbial networks, corresponding to the notion of keystone species (Han et al., 2022). Our findings agree with observations from recent reports (Crump et al., 2009; Wilhelm et al., 2014; de Cena et al., 2021) highlighting the role of low-abundance taxa in ecosystem function and structure. For example, de Cena et al. recently hypothesised that low-abundance taxa, albeit in the human microbiome, act as keystone taxa, and might often be more metabolically influential within the community (de Cena et al., 2021). Similarly, Crump et al. (Crump et al., 2009) identified microbial keystone taxa, albeit neither rare nor abundant, that are central to ecosystem-level metabolic activity. Additionally, as Ren et al. (Ren et al., 2021) highlighted, there may be several causes including but not limited to environmental variables and/or the metacommunity structure of tributaries in Otemma. It is plausible that both metacommunity structure such as connectivity, directionality of dispersal, and also if these sites are rather discrete communities or part of a gradient/continuum may be contributing to these differences (Leibold and Mikkelson, 2002). It is also likely that the physical heterogeneity of the floodplains, i.e. tributaries in Otemma were larger compared to Val Roseg, may potentially lead to greater heterogeneity and thus increased fragmentation.

Work on multi-trophic food webs (Stouffer and Bascompte, 2011) and agroecosystems (Pocock et al., 2012) has demonstrated the fragility of ecological networks towards removal of key nodes. Our fragmentation analysis substantiates the notion of keystone taxa and their role for the stability of the cross-domain network. Interestingly, we identified several eukaryotes as keystone taxa, underscoring their relevance for biofilm structure and functioning. In GFS in Central Asia, Ren et al. (Ren and Gao, 2019) reported that fungi form integral components of cross-domain interactions networks, forming more clustered networks less susceptible to disturbances. As highlighted previously for stream biofilms (Wagner et al., 2015; Busi et al., 2022), eukaryotic algae serve as sources of organic matter thereby fuelling phototrophic-heterotrophic interactions. Simultaneously, parasitic fungi also foster the release of organic compounds from algae via the ‘fungal shunt’ (Klawonn et al., 2021). The prevalence of parasitic fungi has been noted previously in GFS (Kohler et al., 2022) and other cryospheric ecosystems (Anesio et al., 2017); our analyses further point to the importance of interactions among parasitic fungi and their algal host in proglacial stream biofilms. Along these lines, Mo et al. (Mo et al., 2021) recently suggested that interactions of microeukaryotes between them in the Lena River continental shelf were more stable compared to that of the estuary, potentially explained by variability in salinity. In contrast, Liu and Jiang reported that bacteria-bacteria interactions dominate co-occurrence networks in coastal sea waters of Antarctica (Liu and Jiang, 2020) and related this to competitive abilities of prokaryotes.

Taken together, the roles of bacterial and eukaryotic keystone taxa in ecological networks and their stability may very much be context dependent. We argue that, likely driven by the provisioning of organic matter to heterotrophs, eukaryotic algae and their fungal counterparts play central roles in biofilm interaction networks. However, we quantified the importance of pro- and eukaryotic keystone taxa to overall bacterial community structure and found that the relative abundance of bacterial keystone taxa could explain much of the bacterial community structure. This points towards a hierarchical structuring of interactions among eukaryotic and bacterial members of the biofilm. While eukaryotic primary producers may directly interact with only some bacterial keystone taxa, these bacterial keystone taxa themselves interact, likely via the exchange of secondary metabolites, with a much larger number of bacteria in the biofilm assemblage (Hessler et al., 2023). Such a hierarchical organisation of interactions is likely sensitive to changes in taxa at the base (i.e., the algal primary producers) whereas functional redundancies may dampen the impacts of taxa replacement. This may be particularly relevant in proglacial streams, where reduced light availability due to suspended particles and substrate instability typically inhibit algal growth. The current retreat of glaciers weakens these controls with potential effects on stream microbial communities.

## Data availability statement

The datasets presented in this study can be found in online repositories. The names of the repository/repositories and accession number(s) can be found below: https://www.ncbi.nlm.nih.gov/, PRJNA808857 and https://doi.org/10.5281/zenodo.7524289, zenodo-7524289.

## Author contributions

SBB: Conceptualization, Data curation, Formal analysis, Investigation, Methodology, Project administration, Visualization, Validation, Writing – original draft. HP: Conceptualization, Formal analysis, Investigation, Methodology, Writing – original draft, Writing – review & editing. JB: Investigation, Writing – original draft, Writing – review & editing. TK: Writing – original draft, Writing – review & editing. SF: Data curation, Writing – review & editing. PP: Methodology, Writing – review & editing. MB: Data curation, Writing – review & editing. GM: Data curation, Writing – review & editing. LE: Data curation, Writing – review & editing. SL: Conceptualization, Funding acquisition, Resources, Writing – original draft, Writing – review & editing. PW: Conceptualization, Funding acquisition, Project administration, Resources, Supervision, Writing – original draft, Writing – review & editing. TB: Conceptualization, Formal analysis, Funding acquisition, Investigation, Methodology, Project administration, Resources, Supervision, Validation, Writing – original draft, Writing – review & editing.

## Supplementary material

Supplementary Figure 1 16S and 18S community profiles. **(A)** Bird’s eye-view of the Otemma (left) and Val Roseg (right) floodplains depicting the glacier-fed stream mainstem (GFS) and the branching non-glacial tributaries (TRIB). The dashed line indicates the year 2000, where samples were classified as ‘pre-2000 or UPor ‘post-2000 or DOWN’ site above and below, respectively. **(B)** Family-level profiles of the top 15 bacteria found in the floodplains across reaches and stream types (GFS and TRIB). **(C)** Relative abundance of the top 15 eukaryotic photoautotrophs.

Supplementary Figure 2 Taxa contributing to cross-domain interactions in Otemma. Relative abundance of bacteria found in the cross-domain networks of the **(A)** GFS and **(B)** TRIB in Otemma. **(C)** and **(D)** show the relative abundance of the eukaryotic photoautotrophs in the GFS and TRIB respectively, while **(E)** and **(F)** depict the relative abundance of the fungi in Otemma.

Supplementary Figure 3 Taxa contributing to cross-domain interactions in Val Roseg. Relative abundance of bacteria found in Val Roseg in the cross-domain networks of the **(A)** GFS and **(B)** TRIB. Phototroph relative abundances in the **(C)** GFS and **(D)** TRIB. (E) and (F) depict the relative abundance of the fungi in GFS and TRIB in Val Roseg.

Supplementary Figure 4 Keystonetaxa in Otemma. The keystone taxa for the GFS at the **(A)** pre-2000and **(B)** post-2000 reaches are highlighted based on their domain of origin. Keystone taxa in the TRIB at the **(C)** pre-2000and **(D)** post-2000reaches from the TRIB are simultaneously shown. The x-axis represents the overall betweenness of the individual taxa, whereas the y-axis indicates the degree centrality.

Supplementary Figure 5 Keystone taxa in Val Roseg. The keystone taxa for the GFS at the **(A)** pre-2000and **(B)** post-2000reaches in Val Roseg are highlighted. keystone taxa in the tributaries at the **(C)** pre-2000and **(D)** post-2000reaches from the TRIB are depicted in the scatter plots. The x-axis represents the overall betweenness of the individual taxa, whereas the y-axis indicates the degree centrality, i.e., number of connections per node.

Supplementary Table 1 Metadata and network topology. Glacier metadata, including the glacier from which samples were collected, pre-2000or post-2000 reaches, and type of stream, i.e., Glacier-fed (GFS) or tributaries (TRIB), are indicated along with network topology measures. The dashed line (- - - -) indicates the ‘millennium cut’ based on which samples were classified as ‘pre-2000 or up’ or ‘post-2000 or down’. The solid lines represent the deglaciated history based on the Glacier Extent Database to determine the date since ‘last glaciation’.

Supplementary Table 2 Chlorophyll-*α* measurements. Levels of chlorophyll-*α* measured at the site for each sample are listed along with metadata.

Supplementary Table 3 Filtering thresholds for network analyses. ASVs were filtered at various thresholds, i.e. 1%, 5% and 10%, to reduce computational burden without losing diversity. Table indicates ASVs retained at chosen thresholds.

Supplementary Table 4 Pro- and eukaryotic keystone taxa. Abundance information for all ASVs detected in the bacteria (16S) and eukaryote (18S) datasets are provided alongside their indication of Keystone taxa or otherwise.
