# Supplementary figures and images for: Cross-domain interactions confer stability to benthic biofilms in proglacial streams
Source: Front Microbiomes. 2024 Jan 11;2:1280809. doi: 10.3389/frmbi.2023.1280809 (PMC12993512; doi:10.3389/frmbi.2023.1280809)

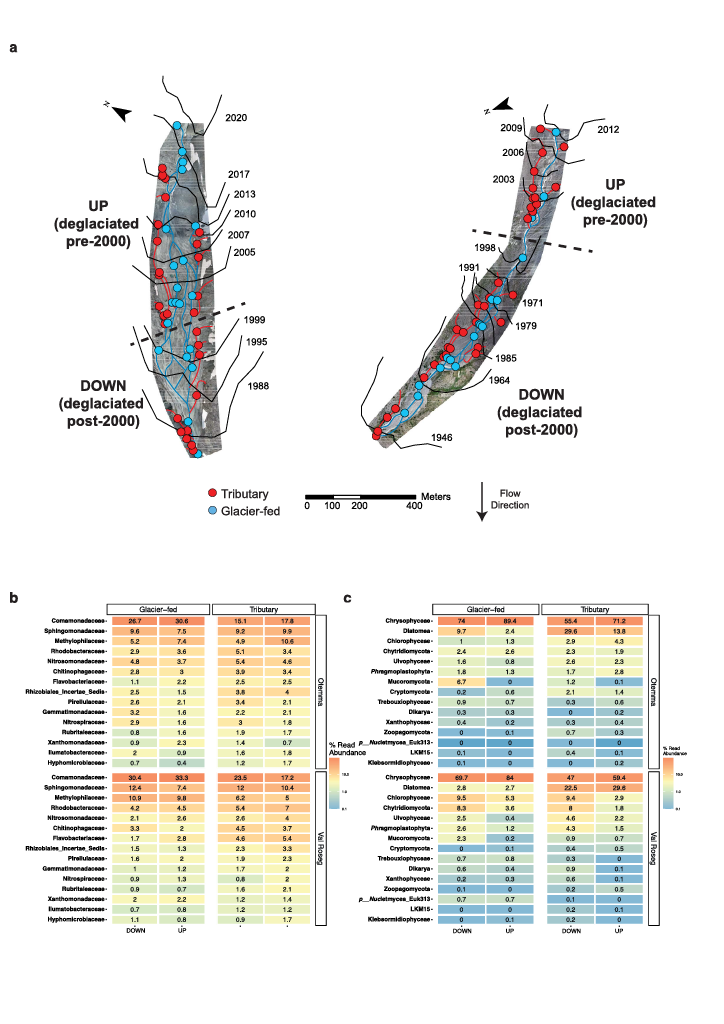

Supplement: Supplementary Figure 1 — 16S and 18S community profiles. (A) Bird’s eye-view of the Otemma (left) and Val Roseg (right) floodplains depicting the glacier-fed stream mainstem (GFS) and the branching non-glacial tributaries (TRIB). The dashed line indicates the year 2000, where samples were classified as ‘pre-2000 or UPor ‘post-2000 or DOWN’ site above and below, respectively. (B) Family-level profiles of the top 15 bacteria found in the floodplains across reaches and stream types (GFS and TRIB). (C) Relative abundance of the top 15 eukaryotic photoautotrophs. [file Image_1.tiff]

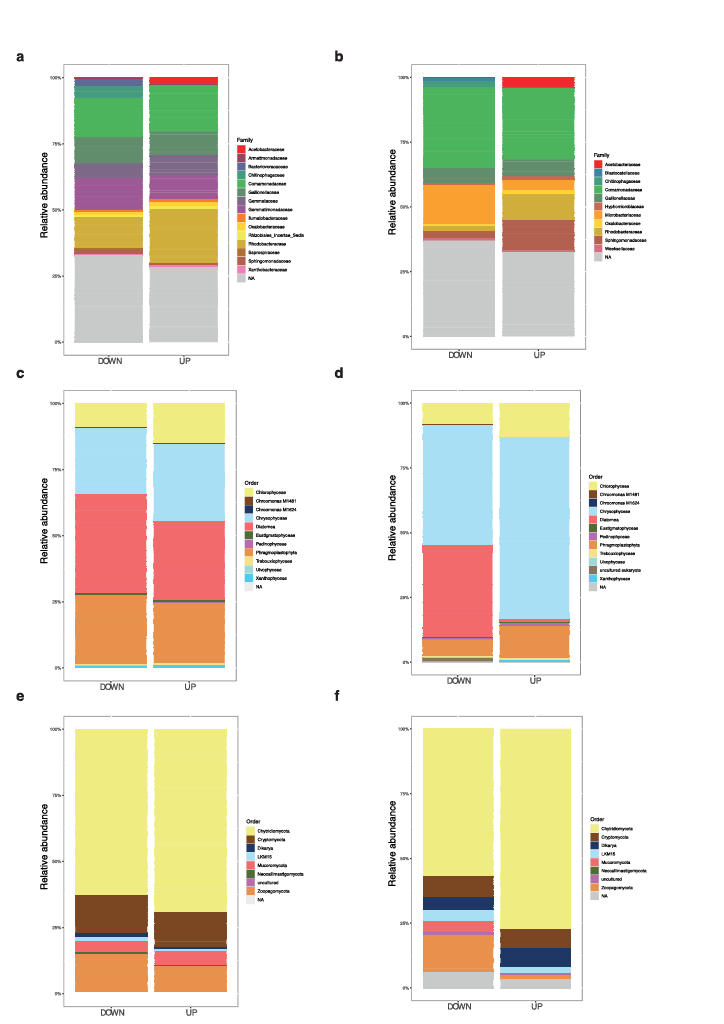

Supplement: Supplementary Figure 2 — Taxa contributing to cross-domain interactions in Otemma. Relative abundance of bacteria found in the cross-domain networks of the (A) GFS and (B) TRIB in Otemma. (C) and (D) show the relative abundance of the eukaryotic photoautotrophs in the GFS and TRIB respectively, while (E) and (F) depict the relative abundance of the fungi in Otemma. [file Image_2.tiff]

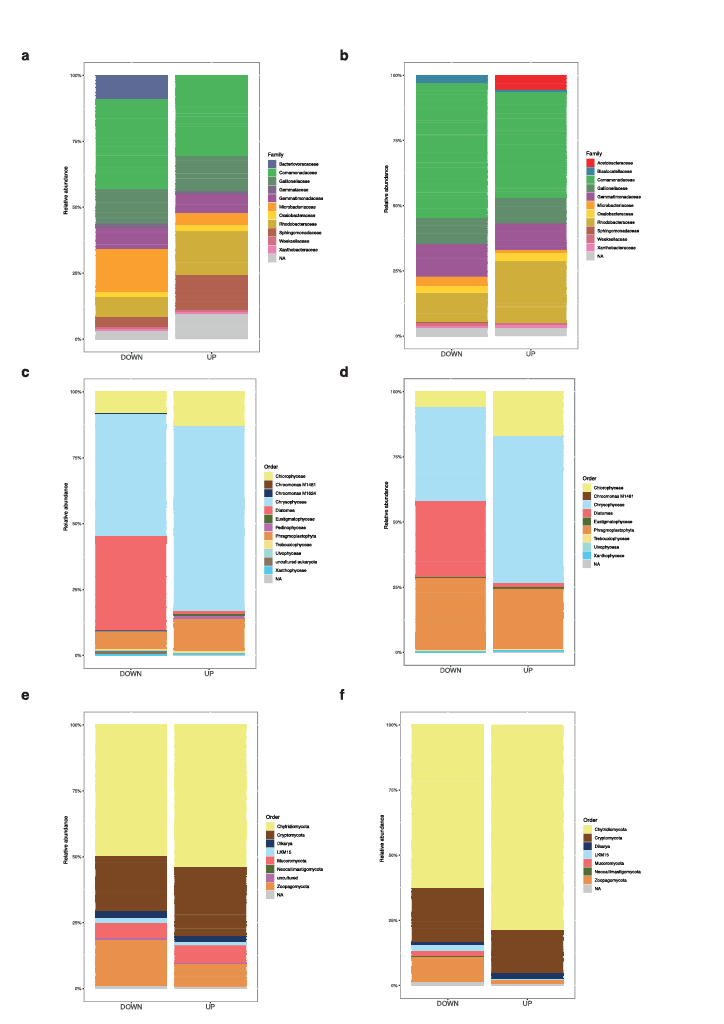

Supplement: Supplementary Figure 3 — Taxa contributing to cross-domain interactions in Val Roseg. Relative abundance of bacteria found in Val Roseg in the cross-domain networks of the (A) GFS and (B) TRIB. Phototroph relative abundances in the (C) GFS and (D) TRIB. (E) and (F) depict the relative abundance of the fungi in GFS and TRIB in Val Roseg. [file Image_3.tiff]

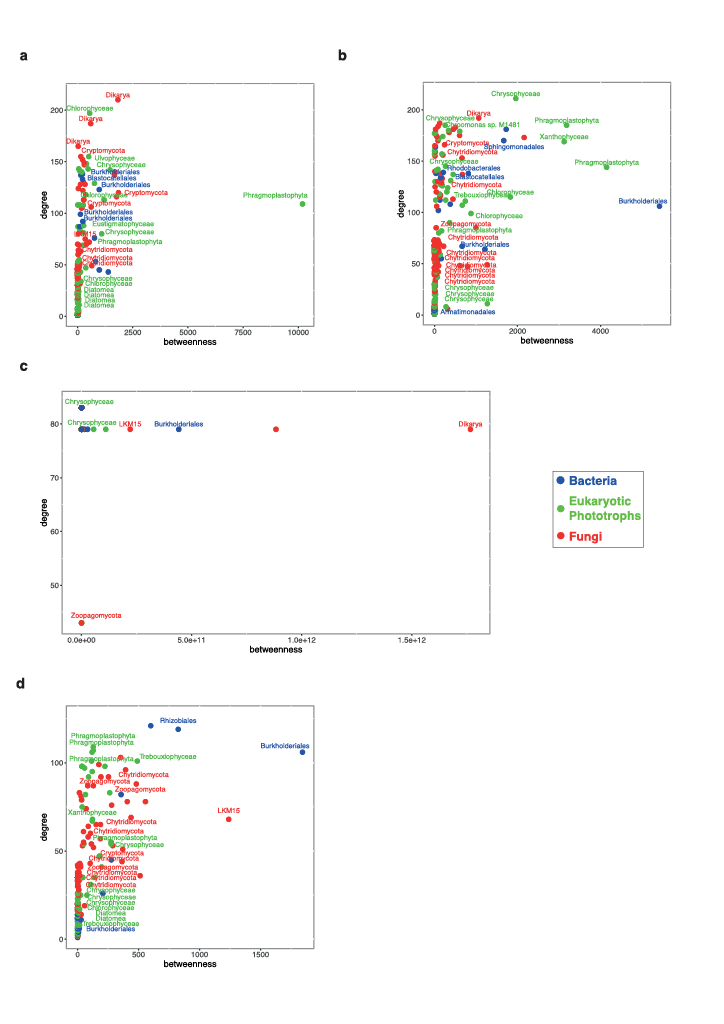

Supplement: Supplementary Figure 4 — Keystonetaxa in Otemma. The keystone taxa for the GFS at the (A) pre-2000and (B) post-2000 reaches are highlighted based on their domain of origin. Keystone taxa in the TRIB at the (C) pre-2000and (D) post-2000reaches from the TRIB are simultaneously shown. The x-axis represents the overall betweenness of the individual taxa, whereas the y-axis indicates the degree centrality. [file Image_4.tiff]

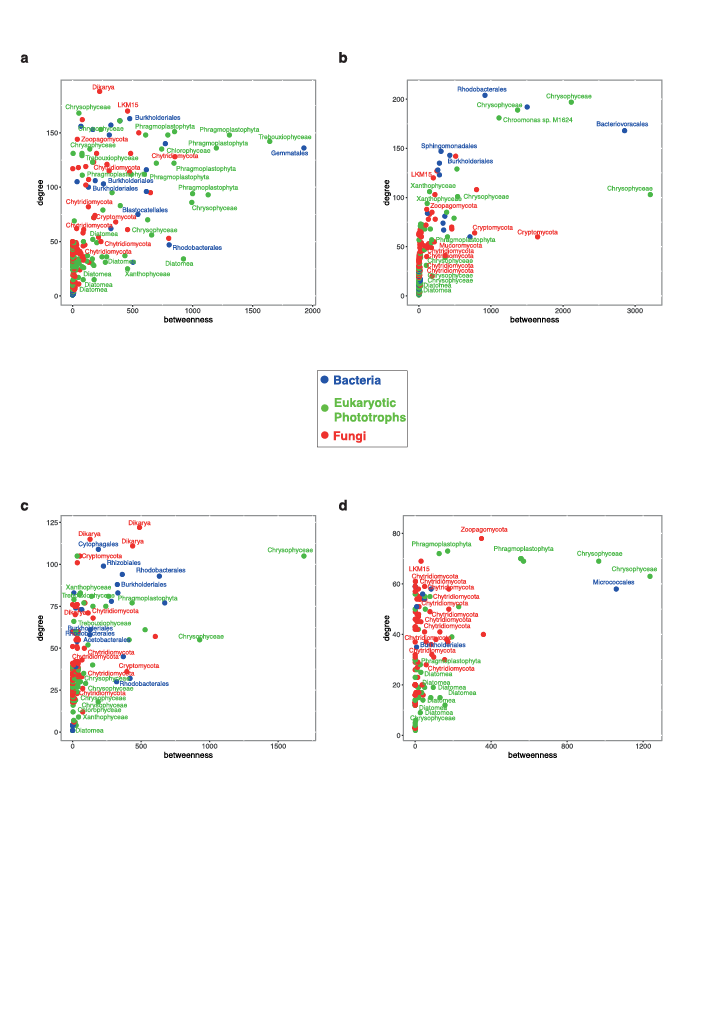

Supplement: Supplementary Figure 5 — Keystone taxa in Val Roseg. The keystone taxa for the GFS at the (A) pre-2000and (B) post-2000reaches in Val Roseg are highlighted. keystone taxa in the tributaries at the (C) pre-2000and (D) post-2000reaches from the TRIB are depicted in the scatter plots. The x-axis represents the overall betweenness of the individual taxa, whereas the y-axis indicates the degree centrality, i.e., number of connections per node. [file Image_5.tiff]
